# Relationship Between Cigarette Smoking and Awake Bruxism: Does Smoking Increase the Frequency of Masticatory Muscle Activities?

**DOI:** 10.1111/joor.13947

**Published:** 2025-01-31

**Authors:** Ovidiu Ionut Saracutu, Matteo Pollis, Alessandro Bracci, Marco Ferrari, Daniele Manfredini

**Affiliations:** ^1^ Department of Medical Biotechnologies, School of Dentistry University of Siena Siena Italy; ^2^ Department of Neurosciences, School of Dentistry University of Padova Padova Italy

**Keywords:** anxiety, awake bruxism, correlation, depression, psyche, smoking

## Abstract

**Background:**

Despite the aetiology of awake bruxism (AB) being prevalently linked to psychological factors, several studies suggested that the use of certain substances, such as tobacco smoking, can contribute to the increase in masticatory muscle activities (MMA) during wakefulness.

**Objective:**

The aim of this study is to assess whether there is a correlation between the frequency of awake bruxism behaviours and smoking habits.

**Methods:**

Participants were recruited, without gender or ethnic restriction, at the University of Siena, Siena, Italy, by advertising. Participants completed a questionnaire containing the four‐item patient health questionnaire for anxiety and depression (PHQ‐4) and some items from the Global Adult Tobacco Smoking (GATS) questionnaire. Moreover, they performed one week of awake bruxism behaviours monitoring via the ecological momentary assessment (EMA).

**Results:**

A total of 100 participants (university employees, dentists, undergraduate and post‐graduate students) were included in the study (34 males and 66 females, mean age 24.5 years). Of them, 39% were smokers and 61% were non‐smokers. The multiple variable linear regression analysis results showed a statistically significant correlation between the frequency of awake bruxism behaviours and the PHQ‐4 scores. Specifically, for every 1% increase in PHQ‐4 score, the mean frequency of the AB behaviours increases 5‐fold. Awake bruxism behaviours did not show any statistically significant correlation with the number of smoked cigarettes (*p* > 0.05). Mandible bracing significantly correlated with the number of years of smoking (*B* = 1.58, *p* = 0.002).

**Conclusions:**

According to the present study's findings, the frequency of awake bruxism behaviours correlated with symptoms of anxiety and depression but not with smoking status.

## Graphical abstract

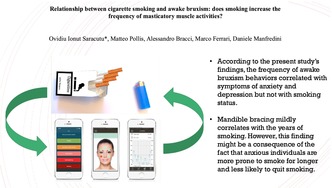

## 1 Introduction

In the 2013 international consensus paper on bruxism, bruxism has been defined as a *repetitive masticatory muscle activity* (*MMA*) *characterised by clenching or grinding of the teeth and/or by bracing or thrusting of the mandible*. In the paper, it was also stated that bruxism can have two circadian manifestations: one during wakefulness, i.e., awake bruxism (AB), and one during sleep, i.e., sleep bruxism (SB) [1]. In 2018, a second consensus paper further clarified and provided two separate definitions for bruxism. Awake bruxism was defined as *a masticatory muscle activity during wakefulness that is characterised by repetitive or sustained tooth contact and/or by bracing or thrusting of the mandible and is not a movement disorder in otherwise healthy individuals*, while sleep bruxism as a *masticatory muscle activity during sleep that is characterised as rhythmic* (*phasic*) *or non‐rhythmic* (*tonic*) *and is not a movement disorder or a sleep disorder in otherwise healthy individuals* [2].

A secondary goal of the paper was to clarify that bruxism should not be considered a disorder, but rather assessed as a sign associated with certain underlying habits and conditions as well as some potential clinical consequences. Indeed, high masticatory muscle activity can have negative outcomes such as severe masticatory muscle pain, mechanical fatigue to tooth and restorations and prosthodontic complications [3, 4, 5, 6, 7]. Still, it could also be a protective factor for obstructive sleep apnea [8] and gastroesophageal reflux disease (GERD) [9]. Given the potentially equivocal interpretation of the terms ‘*otherwise healthy individuals*’, five years later, some of the authors of the consensus paper on bruxism wrote an explanatory note to clarify that in no circumstances could bruxism be seen as ‘the’ disorder, being rather a sign of an underlying or associated condition [10].

From the consensus papers, the need to standardise the assessment of bruxism also emerged, which led to the development of the first multidimensional Standardised Tool for the Assessment of Bruxism (STAB) [11, 12, 13]. The STAB consists of an Axis A for the evaluation of bruxism status and potential consequences based on the different methods available, the subject based (self‐report), the clinically based (clinical examination) and instrumentally based ([e.g., Ecological Momentary Assessment (EMA), wake‐time and night‐time electromyography and polysomnography]) assessment, and an Axis B evaluating risk factors and co‐occurring underlying conditions (e.g., anxiety and depression, gastroesophageal reflux disease and orofacial motor disorders).

As far as awake bruxism is concerned, its aetiology is likely multifactorial, albeit prevalently linked to psychological issues. In this regard, while some studies revealed a genetic component [14, 15], most investigations confirmed the determinant role of psychological factors [16]. Studies that evaluated bruxism by means of a questionnaire found a statistically significant association between awake bruxism and anxiety [17], mood disorders [18] and depressive and manic symptoms [19]. The same associations were confirmed by more elaborated strategies for the assessment of awake bruxism, such as the EMA [20, 21]. A recent cross‐sectional study that adopted the same approach found that awake bruxism behaviours, such as mandible bracing, teeth contact and teeth clenching, are significantly more prevalent in individuals with anxiety and depression traits [22].

Additionally, some authors suggested that the use of certain substances, such as tobacco smoking, has also been positively associated with an increase in the MMA [23, 24, 25], even if one study did not find any association [26]. Most studies focused their attention on the specific relationship between sleep bruxism and smoking, excluding awake bruxism behaviours from the analysis [27, 28]. Indeed, up to now, no study has tried to unveil if there is a dose–response relationship between tobacco smoking and awake bruxism behaviour frequency, and most of them are only based on self‐reported awake bruxism, with a possible investigation bias associated.

In the same way, the influence of psychological factors on the relationship between bruxism and tobacco smoking is far from being completely understood. Indeed, smokers generally report that cigarette smoking helps them to relieve stress [29, 30], and there are also suggestions on the potential anxiolytic effect of smoking [31]. Conversely, long‐term chronic tobacco smoking seems to be associated with an increase in anxiety and depression symptoms [32, 33, 34]. Evidence deriving from experimental models suggests that nicotine can exert its effect on the mesolimbic dopamine system and hippocampus, areas of the brain involved in anxiolysis and anxiogenesis. Following chronic use, a molecular and cellular adaptation to nicotine occurs in the brain, with a consequence increase in anxiety and depression during tobacco abstinence [32].

Within this scenario, it can be hypothesized that, given the relationship of long‐term smoking with anxiety and depression, and given the importance of these psychological factors in the aetiology of AB, chronic smoking might contribute to the increased level of AB indirectly.

To gather data to start testing this hypothesis, a cross‐sectional investigation was performed in a sample of healthy young adults, creating for the first time a multiple‐variable model to predict the influence of the number of cigarettes smoked, the years of smoking and the psychological status on the frequency of awake bruxism behaviours assessed via EMA. This paper reports the results of the investigation.

## 2 Materials and Methods

### 2.1 Participants Recruitment

Participants were recruited, without gender or ethnic restriction, at the University of Siena, Siena, Italy, by advertising. The inclusion criteria were having good general health without any neurological, systemic, autoimmune or oral disease. Exclusion criteria were ongoing medical or dental treatment or having been treated for AB and temporomandibular disorders (TMDs). The TMD Pain screener was administered to rule out TMD patients [35]. Once participants accepted to take part in the study, they signed the informed consent and received a paper with the instructions to follow. In the instruction paper, it was written that participants are required to attend a 2‐h seminar with the leading investigator (A.B.) and the study supervisor (D.M.), where they would be instructed on the use of a smartphone‐based application for the monitoring of awake bruxism behaviours. It was also specified that they would receive a questionnaire to fill out, containing items regarding their psychological status and their smoking habits.

All individuals gave their informed consent in accordance with the Helsinki Declaration and understood that they were free to withdraw from the study at any time. The research protocol was approved by the Institutional Review Board of the Orofacial Pain Unit, University of Siena, Siena, Italy (#0007–2020).

### 2.2 Awake Bruxism Assessment

During the first hour of the seminar, participants were introduced to the new definitions of bruxism and to the types of masticatory muscle activities that can be performed during wakefulness (i.e., relaxed jaw muscle, tooth contact, mandible bracing, teeth clenching and teeth grinding). Afterward, participants received an instruction paper on using a smartphone‐based application BruxApp (BruxApp, World Medical Applications Srl, Italy) for the ecological momentary assessment of AB behaviours frequency (Item A8.1 of the STAB). The app sends 20 alerts with accompanying sounds randomly throughout the day. The app lists masticatory muscle activities specific to awake bruxism behaviours for each alert sound. When the app sends a notification with the alert, the user has 5 min to select the specific condition they are experiencing from the listed options. Any response provided after 5 min will not be recorded as it could be influenced by recall bias. Participants were also instructed to ignore the alert if they heard it while engaging in activities such as speaking, eating or drinking unrelated to awake bruxism. The instruction paper and the questionnaire contained an ID code unique for each participant. The masticatory muscle activities that are listed by the app, with each alert sound, are the following:

1. Relaxed jaw muscle: condition of perceived relax of jaw muscles, with mandibles kept apart;
2. Mandible bracing: condition of jaw muscle stiffness or tension like teeth clenching, but with teeth kept apart;
3. Teeth contact: condition of repetitive slight teeth contact like the teeth contact that the subject perceives when a 40 μ articulating paper (Bausch Occlusionspapier; Bausch KG, Koln, Germany) is put between the dental arches and he/she is asked to slightly keep the teeth in contact to retain it on site;
4. Teeth clenching: all conditions in which teeth contacts are more marked than the above and jaw muscles are kept tense;
5. Teeth grinding: condition in which the opposite teeth are gnashed or ground, independently by intensity and direction of antagonist teeth contacts.

The app was designed to send alerts during specific time slots, from 08:00 to 12:00, 15:00 to 19:00 and 21:00 to 22:00, to minimise the chances of receiving alerts during meals. If, at the end of the assessment period, at least 70% of the alerts were responded to over seven days, the app would generate a report. This meant that participants needed to respond to at least 12 alerts out of 20 programmed each day, totaling 84 out of the 140 received in one week. If this minimum threshold was not met, the app would extend the registration period to a maximum of seven days to ensure that there were at least seven days of assessment with 12 alerts answered per day. Participants were not able to view the results of the 7‐day assessment before the report was generated. After generating the report, Bruxapp would notify the participant to send it via email to the researchers involved in the data collection, along with the ID code provided in the instruction paper. The reports generated contained an anonymous pre‐formatted Excel file, which detailed the number of alerts answered and the frequency of various awake bruxism conditions, such as relaxed jaw muscle, mandible bracing, teeth contact, teeth clenching and teeth grinding. The ID code of the Bruxapp report was then matched with the corresponding questionnaires that contained only the ID code and no other information that would allow the identification of the participants. These AB frequencies were calculated as a percentage of the total alerts answered. The file included the daily frequency of each condition on an individual basis and the frequency of all conditions over the seven valid days of assessment.

### 2.3 Psychological Assessment

In the second part of the meeting, the participants were asked to fill out a printed questionnaire. The first part of the questionnaire contained the four‐item Patient Health Questionnaire for anxiety and depression (PHQ‐4), item B1.1 of the STAB. The questionnaire contains four items for the screening of anxiety and depression:

*Over the last two weeks, how often have you been bothered by the following problems?*

1. Feeling nervous, anxious, or on edge;
2. Not being able to stop or control worrying;
3. Feeling down, depressed, or hopeless;
4. Little interest or pleasure in doing things.

For each item, the participant could attribute a score representing the frequency of the problem: Not at all = 0, Several days = 1, More than half the days = 2, Nearly every day = 3. The total score of the questionnaire ranges from 0 to 12, and according to it, participants can be divided into four groups: group 1 normal (0–2), group 2 mild (3–5), group 3 moderate (6–8) and group 4 severe (9–12).

### 2.4 Smoking Status

The questionnaire for smoking status contained the following three questions taken from the Global Adult Tobacco Smoking (GATS) questionnaire [36]:

1. Do you *currently* smoke tobacco on a daily basis, less than daily, or not at all? (Item B01 of the GATS)
  - Daily…
  - Less than daily…
  - Not at all…
  - Don't know…
2. On average, how many manufactured cigarettes do you currently smoke each week? (Item B06, a1 of the GATS)
3. On average, how many hand‐rolled cigarettes do you currently smoke each week? (Item B06, b1 of the GATS)
4. On average, how many pipes full of tobacco do you currently smoke each week? (Item B06, d1 of the GATS)
5. On average, how many cigars, cheroots, or cigarillos do you currently smoke each week? (Item B06, e1 of the GATS)
6. How many years ago did you first start smoking tobacco *daily*? (Item B05 of the GATS)
7. In the *past*, have you smoked tobacco on a daily basis, less than daily, or not at all? (Item B03 of the GATS)
  - Daily…
  - Less than daily…
  - Not at all…
  - Don't know…
8. How many years ago did you first start smoking tobacco *daily*? (Item B12 of the GATS)

After the end of the AB monitoring via EMA, the data were matched with the questionnaires through the ID code.

### 2.5 Statistical Analysis

Data were extracted by two independent reviewers (O.I.S and M.P.) and put into an Excel document. SPSS 26.0 (SPSS Inc., Chicago, USA) was used to perform the statistical analysis. A descriptive analysis of each awake bruxism behaviour was performed. Data were reported as mean values, standard deviation (SD) and coefficient of variation (CV). A multiple variable linear regression with the backward model was created, keeping awake bruxism behaviours frequency as the dependent variable, while psychological distress, number of cigarettes and years of smoking were considered independent variables. The endpoint data were evaluated using the 95% confidence interval (CI). The level of significance was set at *p* < 0.05.

## 3 Results

**FIGURE 1:**
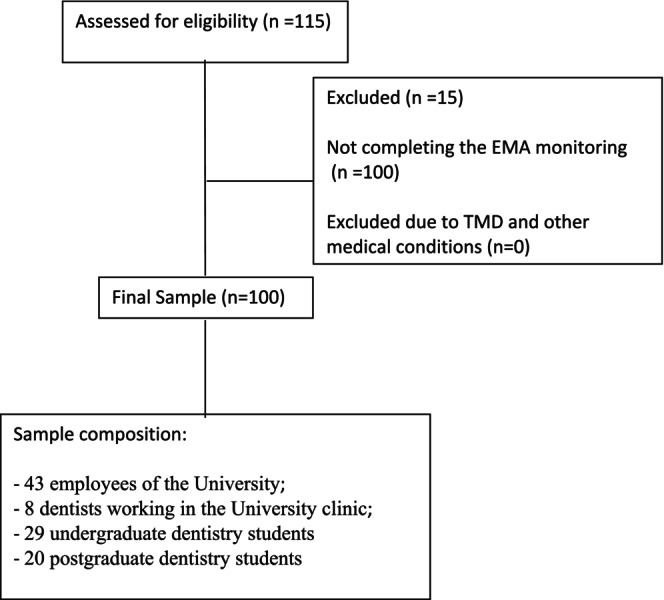
Flow‐chart showing the enrollment process of participants.

A total of 115 people showed interest in participating in the investigation and attended the 2‐h seminar. Of them, 15 participants (13%) did not complete the 7‐day monitoring period for the assessment of AB. A total of 100 participants (87% of the total) were included in the study (34 males and 66 females, mean age 24.5 years, range 19–32) (Figure 1). The final sample was composed of 43 employees of the University, 8 dentists working in the University clinic, 29 undergraduate dentistry students and 20 postgraduate dentistry students. Concerning the compliance of EMA of AB, the average response rate was 73.3% ± 7.1% (Table 1).

**TABLE 1:** Mean and standard deviation (SD) for alert response rate for each day, the total mean and the SD.

Alert response rate	D1	D2	D3	D4	D5	D6	D7	Mean of confirmed alerts
Mean	71.5%	72.1%	75.9%	73.3%	74.0%	74.9%	72.3%	73.3%
SD	11.5%	12.3%	11.4%	15.6%	12.3%	12.5%	13.4%	7.1%

*Note:* Mean frequency value is the number of positive responses for each day. For instance, a mean of 74.5% for D1 can be interpreted as the equivalent of answering to 74.5% of the 20 pre‐set alerts.

Abbreviations: D, day; SD, standard deviation.

The mean frequency of the various AB‐related behaviours was calculated as follows, in order of frequency: teeth contact (11.8% ± 13.9%); mandible bracing (5.9% ± 11.2%); teeth clenching (1.7% ± 3.5%) and teeth grinding (0.7% ± 0.5%). Teeth contact was the most reported AB behaviour, whilst relaxed jaw muscles was reported in 79.2% ± 21.1% of the answers. The coefficient of variation CV is expressed as the ratio between SD and mean for each of the seven days of AB assessment (Table 2).

**TABLE 2:** Frequency data in percentage (mean, standard deviation, coefficient of variation) for the AB behaviours during the 7‐days of observation.

Activity	Mean	SD	CV	Range
Relaxed jaw muscles	79.2%	22.1%	0.3%	5.2%–100%
Teeth contact	11.8%	13.9%	1.2%	0%–80%
Mandible bracing	5.9%	11.2%	1.9%	0%–72.5%
Teeth clenching	1.7%	3.5%	2.1%	0%–28.2%
Teeth grinding	0.7%	0.5%	0.7%	0%–3.3%

*Note:* For each AB behaviour, mean frequency value is number of positive responses in % of the total amount of alert to which the participant responded. For instance, a mean of 79.2% for the ‘Relaxed Jaw Muscles’ condition, can be interpreted as equivalent to the report of 79.2% ‘Relaxed Jaw Muscles’ per 100 reporting alerts, meaning that on average, each subject answered Relaxed Jaw Muscles to 79.2% of the alerts, generalising from the random EMA sampling, with a minimum of 5.2% by at least one subject and a maximum of 100% by at least one subject.

Abbreviations: CV, coefficient of variation; D, Day; SD, standard deviation.

Table 3 reports the mean frequency of the PHQ‐4 psychological status for the studied sample. According to the PHQ‐4 score, more than 50% of participants (*N* = 59) had a normal status for depression and anxiety. Conversely, 41% of the study population had had a PHQ‐4 score ≥ 3.

**TABLE 3:** PHQ‐4 status of the study population.

Depression and anxiety status	PHQ‐4
0–2 = normal	59% (*N* = 59)
3–5 = mild	13% (*N* = 13)
6–9 = moderate	27% (*N* = 27)
10–12 = severe	1% (*N* = 1)

Of the total amount of participants, 39% were smokers, and 61% were non‐smokers, while no former smoker was present. The tobacco smokers present in the current study only smoked manufactured cigarette smoking (74% of smokers) and hand‐rolled cigarettes (26% of smokers). The average number of daily smoked cigarettes was 6 ± 3.65, ranging from 2 to as high as 14. The average number of years of daily smoking was 3.7 ± 1.96 years, ranging from 1 to 8 (Table 4). No statistically significant difference was found between students and non‐students regarding the frequency of AB behaviour and the distribution of the PHQ‐4 (*p* > 0.05).

**TABLE 4:** Smoking status of the study population.

	Mean	SD	Range
Smokers	39		
Non‐smokers	61		
Former smokers	0		
Number of cigarettes smoked daily	6	3.65	2–14
Number of years of daily smoking	3.7	1.96	1–8

The multiple variable linear regression analysis results showed a statistically significant correlation between the frequency of awake bruxism behaviours and the PHQ‐4 scores. Relaxed jaw muscles showed a strong negative correlation with anxiety and depression status (*B* = −5.32, *p* < 0.001). Specifically, for every 5.32% decrease in the frequency of relaxed condition there was a 1% increase in PHQ‐4 score. Conversely, tooth contact (*B* = 2.78, *p* > 0.001), mandible bracing (*B* = 1.7, *p* < 0.001) and teeth clenching (*B* = 0.7, *p* < 0.001) showed a positive correlation with the PHQ‐4 scores. No correlation was found between psychological status and teeth grinding. A strong correlation was found between the sum of all the awake bruxism behaviours and the PHQ‐4 scores (*B* = 5.37, *p* < 0.001). No awake bruxism behaviour showed any statistically significant correlation with the number of smoked cigarettes (*p* > 0.05). Mandible bracing significantly correlated with the number of years of smoking (*B* = 1.58, *p* = 0.002). The other types of AB behaviours did not significantly correlate with the number of years of smoking. Table 5 shows the results of the multiple linear regression analysis.

**TABLE 5:** Results of the multiple linear regression analysis with backward model.

Dependent variable	Independent variable	Unstandardized coefficients		Standardised coefficient	t	*P*	95% confidence interval for B	
		B	Std. error	Beta		Sig.	Lower bound	Upper bound
Relaxed jaw muscle	Constant	90.923	2.106		43.18	< 0.001^a^	86.743	95.103
	PHQ‐4	−5.123	0.659	−0.684	−7.771	< 0.001	−6.432	−3.814
	Number of cigarettes	−0.135	0.585	−0.024	−0.23	0.819^b^	−1.296	1.026
	Years of smoking	−0.935	1.007	−0.083	−0.928	0.356^b^	−2.935	1.065
	Constant	90.952	2.092		43.485	< 0.001	86.801	95.104
	PHQ‐4	−5.21	0.537	−0.696	−9.704	< 0.001^a^	−6.276	−4.145
	Years of smoking	−1.073	0.807	−0.095	−1.329	0.187^b^	−2.674	0.529
	Constant	89.987	1.969		45.698	< 0.001	86.079	93.895
	PHQ‐4	−5.319	0.533	−0.71	−9.982	< 0.001^a^	−6.376	−4.261
Tooth contact	Constant	7.23	1.911		3.783	< 0.001	3.436	11.024
	PHQ‐4	2.972	0.598	0.539	4.966	< 0.001^a^	1.784	4.16
	Number of cigarettes	−0.199	0.531	−0.049	−0.375	0.708^b^	−1.253	0.854
	Years of smoking	−0.455	0.914	−0.055	−0.498	0.62^b^	−2.27	1.36
	Constant	7.273	1.899		3.829	< 0.001	3.504	11.043
	PHQ‐4	2.843	0.488	0.515	5.831	< 0.001^a^	1.875	3.811
	Years of smoking	−0.659	0.733	−0.079	−0.899	0.371^b^	−2.113	0.795
	Constant	6.68	1.779		3.754	< 0.001	3.149	10.212
	PHQ‐4	2.776	0.481	0.503	5.767	< 0.001^a^	1.821	3.732
Mandible bracing	Constant	1.069	1.266		0.844	0.401	−1.444	3.582
	PHQ‐4	1.559	0.396	0.411	3.934	< 0.001^a^	0.772	2.346
	Number of cigarettes	0.195	0.352	0.07	0.554	0.581^b^	−0.503	0.893
	Years of smoking	1.386	0.606	0.243	2.288	0.024^b^	0.184	2.588
	Constant	1.027	1.259		0.815	0.417^b^	−1.473	3.526
	PHQ‐4	1.685	0.323	0.444	5.214	< 0.001^a^	1.044	2.327
	Years of smoking	1.585	0.486	0.278	3.263	0.002^a^	0.621	2.549
Teeth clenching	Constant	0.743	0.575		1.292	0.199	−0.399	1.885
	PHQ‐4	0.632	0.18	0.393	3.511	< 0.001^b^	0.275	0.99
	Number of cigarettes	0.137	0.16	0.116	0.859	0.392^b^	−0.18	0.454
	Years of smoking	−0.04	0.275	−0.017	−0.145	0.885^b^	−0.586	0.506
	Constant	0.717	0.543		1.32	0.19	−0.361	1.795
	PHQ‐4	0.639	0.174	0.397	3.673	< 0.001^b^	0.294	0.984
	Years of smoking	0.123	0.128	0.104	0.965	0.337^b^	−0.13	0.377
	Constant	0.803	0.535		1.501	0.137	−0.259	1.866
	PHQ‐4	0.731	0.145	0.454	5.048	< 0.001^a^	0.444	1.019
Teeth grinding	Constant	0.292	0.167		1.748	0.084	−0.04	0.623
	PHQ‐4	−0.025	0.052	−0.06	−0.486	0.628^b^	−0.129	0.078
	Number of cigarettes	0.064	0.046	0.207	1.387	0.169^b^	−0.028	0.156
	Years of smoking	0.007	0.08	0.01	0.083	0.934^b^	−0.152	0.165
	Constant	0.296	0.158		1.879	0.063	−0.017	0.609
	PHQ‐4	−0.026	0.05	−0.063	−0.524	0.602^b^	−0.127	0.074
	Years of smoking	0.067	0.037	0.215	1.793	0.076^b^	−0.007	0.14
	Constant	0.26	0.141		1.842	0.069	−0.02	0.54
	PHQ‐4	0.056	0.031	0.18	1.811	0.073^b^	−0.005	0.117
Sum of all awake bruxism behaviours	Constant	9.3	2.166		4.294	< 0.001	5.001	13.6
	PHQ‐4	5.13	0.678	0.672	7.565	< 0.001^a^	3.784	6.477
	Number of cigarettes	0.203	0.602	0.036	0.337	0.737^b^	−0.991	1.397
	Years of smoking	0.885	1.036	0.077	0.854	0.395^b^	−1.172	2.942
	Constant	9.256	2.152		4.301	< 0.001	4.985	13.528
	PHQ‐4	5.262	0.552	0.689	9.524	< 0.001^a^	4.165	6.358
	Years of smoking	1.093	0.83	0.095	1.316	0.191^b^	−0.555	2.74
	Constant	10.24	2.026		5.054	< 0.001	6.219	14.26
	PHQ‐4	5.372	0.548	0.704	9.8	< 0.001^c^	4.284	6.46

Abbreviation: PHQ‐4, patient health related questionnaire 4.

^a^ Significant at 0.001.

^b^ Non‐significant.

^c^ Significant at 0.5.

## 4 Discussion

The aim of this cross‐sectional investigation was to assess the correlation between the frequency of awake bruxism behaviours with psychological status and smoking habits. A multiple‐variable model was created to study the relationship between the variables. The statistical analysis results show a statistically significant association between the psychological status and the mean frequency of AB behaviours. Namely, for every 1% increase in PHQ‐4 score, the mean frequency of the AB behaviours increases 5‐fold. Tooth contact was the condition that most correlated with anxiety and depression symptoms. In the study sample of healthy young individuals, the number of daily smoked cigarettes did not correlate with any AB behaviour, while the years of smoking (i.e., history of smoking) mildly correlated only with the frequency of mandible bracing. The possible explanation for such a finding could rely on the effect of smoking on skeletal muscles. It has been shown that smoking increases the ability to voluntarily contract muscles due to the increase in sympathetic nerve activity [37].

The included population consisted of young adults without systemic diseases. To reduce the risk that other comorbidities can influence the frequency of AB behaviours [13], it was decided not to include people affected by systemic or psychiatric conditions. Moreover, the capability of individuals with psychiatric conditions to use smartphone‐based applications could be impaired. Consequently, the study sample consisted of a specific group of young adults with an average age of 24.5 years.

The frequency of AB behaviours was assessed via the EMA. This approach allows clinicians and researchers the possibility to monitor, quantify and qualify the AB behaviours occurring during the day. Several studies on the EMA of awake bruxism have been published in the literature [21, 38, 39, 40, 41, 42, 43, 44, 45, 46, 47]. The approach was included in the STAB as one of AB's main instrumental assessment strategies [13]. The mean frequency of AB behaviours obtained in the present study (i.e., tooth contact 11.8%, mandible bracing 5.9%, teeth clenching 1.7%, teeth grinding 0.7%) is comparable to other investigations performed on healthy individuals [42, 43, 44, 45]. Despite the heterogeneous composition of the sample, no significant difference was found in the frequency of AB behaviours between students and non‐students. Nevertheless, all the individuals included in the study have occupations related to the academic field, which could explain the similar distribution of AB behaviour among the participants.

The psychological status was assessed using the PHQ‐4 questionnaire, which utilises a 0 to 12 scale to screen for potential anxiety and depression. This tool has been validated on a large sample of patients as a general marker for psychological distress [48] and has also been included in the STAB. In the present study, more than one‐third of the sample had a PHQ‐4 score ≥ 3. A more precise psychological assessment could have been performed by integrating the PHQ‐4 into the EMA, given the natural fluctuation of mood disorders and the positive patients' experience of using a dedicated smartphone‐based app [46].

The smoking status was evaluated via a series of items taken from the GATS, a questionnaire created by the World Health Organisation (WHO) to collect data on adult tobacco use [36]. Compared to Item B4.2 of the STAB on tobacco use [13], the selected items of the GATS provide more information, especially concerning the number of years of smoking, which is the number of years a patient was exposed to the risk factor of smoking. Considering that the only correlation concerned the number of years of smoking and mandible bracing, it could be advisable to implement the STAB with a series of items that also assess the number of years a person was exposed to a certain risk factor. This suggestion is in line with the view of the STAB as an open project that can be reviewed from time to time. Another aspect that should be taken into consideration is the distribution of smoked cigarettes during the day, which in the present study was not investigated. It is important to consider that the same number of cigarettes can be smoked within different timeframes. Some individuals consume cigarettes regularly, while others consume only on certain occasions (e.g., social smokers) [49]. Future studies should focus on whether different patterns of smoking are more significantly associated with awake bruxism compared to others. Such a hypothesis could be tested by the combined use of the EMA strategies to assess both AB and smoking behaviour [50].

To the authors' knowledge, this is the first study that tried to correlate the frequency of AB with the smoking status. Therefore, a direct comparison with other similar investigations is not possible. Indeed, even if in the years following the publication of the 2018 consensus paper, there has been an increase in the number of publications on awake bruxism, none of them studied the correlation with smoking. All the literature papers on smoking focused either on the unspecific bruxism report or sleep bruxism.

The first paper on the correlation between SB and tobacco smoking was published by Lavigne et al. in 1997. The main finding was that smokers at night presented five more times episodes of teeth grinding compared to non‐smokers. It is worth mentioning that the approach used to assess bruxism was based on the diagnostic criteria for SB via polysomnography (PSG). Such classification limits the identification of bruxism to those EMG events that are above a specific cut‐off threshold point, generally 10% or 20% of the maximum voluntary contraction (MVC), and are associated with sleep arousals [51]. Such an approach is no longer in line with the current concept of bruxism since it lacks an evaluation of muscle work in its continuum, which can paradoxically be reported subjectively by the patients [52]. More advanced strategies for the measurement of masticatory muscle activity have been included in the STAB [13] with the advent of novel portable electromyographic devices capable of measuring the MMA in its continuum, making use of elaborated outcomes such as the bruxism time index and the bruxism work index [53, 54]. Ahlberg et al. in 2004 analysed the data from a subsample of 1339 employees of a Finnish Broadcasting Company, dividing them into bruxers and non‐bruxers according to the sum of two questions on self‐reported teeth grinding. The total score ranged from 2 to 10, and the authors, in an arbitrary way, indicated 3.4 as the cut‐off value to distinguish frequent bruxers from non‐frequent bruxers [55]. By using such an approach, the authors found a significant association between smoking and frequent bruxers; however, no information on the dose–response relationship was provided, and data on the association between non‐frequent bruxers and smoking were not described. Moreover, the study lacked a psycho‐emotional characterisation of the subjects, which, according to the STAB, is fundamental when performing AB assessment [13]. In 2010, Rintakoski et al. published two cohort studies on a large sample of over 12 000 Finnish twins. In both investigations, the authors based their assessment of bruxism on the questionnaire. The researchers found that smokers are two times more at risk of reporting bruxism. However, in the 2010 study, the strict relationship between smoking and bruxism was put into question, as the authors found that people who made use of smokeless tobacco had a higher odds ratio of reporting bruxism compared to cigarette smoking, thus suggesting that the gesture, which is in turn potentially related with psychological issues, is potentially more important than tobacco as far as the relationship with bruxism is concerned [24, 28]. More than a decade later, the relationship between bruxism and smoking was further put into question when the same authors involved in the previous investigations published a follow‐up of the Finnish Twins Cohort [56]. The prospective study showed that smoking cessation is not associated with a reduction in reports of sleep bruxism.

Within these premises, the relationship between smoking and bruxism remains unclear. The findings of the present study, besides confirming the strong correlation between psyche and AB, do not prove any clear correlation between smoking status and AB behaviour, except for mandible bracing that was mildly correlated with the number of years of smoking. The possible link between mandible bracing and smoking could be related to the repetitive behaviour of pushing the mandible forward to support the cigarette between the lips during smoking. Such behaviour could also remain prevalent during non‐smoking moments. As for the other types of behaviours, it is possible to hypothesize that the most detrimental risk factor for AB remains the psyche and, considering that people with anxiety are more prone to smoke [57, 58], the smoking habit could be seen as an expression of psychological distress [59] that is secondarily related with bruxism report. Indeed, it was shown that people with anxiety disorders are more predisposed to smoke for longer periods of their lives and less prone to quit smoking [60]. The contradictory findings of the previous papers on the association between smoking and bruxism might also be explained by the single variables analysis that has been conducted without taking into consideration the role of the psyche. Another possible reason could be related to the fact that past studies are based only on the self‐report of bruxism [61, 62], with the possible risk of overestimating or underestimating its frequency compared to EMA [63].

To study the relationship between tobacco smoking and bruxism in more detail, further studies should consider the use of novel portable surface electromyographic devices for the 24‐h monitoring of masticatory muscle activity [53], also considering the monitorization of the distribution of smoking during the day and the daily fluctuation of anxiety.

## 5 Conclusion

According to the present study's findings, the frequency of awake bruxism behaviours correlated with symptoms of anxiety and depression but not with smoking status. Mandible bracing mildly correlates with the years of smoking. However, this finding might be a consequence of the fact that anxious individuals are more prone to smoke for longer and less likely to quit smoking.

## Author Contributions

O.I.S. contributed to collection of the data, data processing and writing of the original draft. A.B. conceptualised the study and contributed to data collection. M.F. contributed to data collection and supervised the investigation. M.P. took part in the collection of the data, data processing and editing of the original draft. D.M. has been involved in conceptualization, methodology, supervision, reviewing and editing of the original draft.

## Consent

All individuals gave their informed consent in accordance with the Helsinki Declaration and understood that they were free to withdraw from the study at any time. The research protocol was approved by the Institutional Review Board of the Orofacial Pain Unit, University of Siena, Siena, Italy (#0007–2020).

## Conflicts of Interest

A.B. took part as a shareholder of the WMA srl Company for the development of software for smartphones. The remaining authors declare no conflicts of interest.

### Peer Review

The peer review history for this article is available at https://www.webofscience.com/api/gateway/wos/peer‐review/10.1111/joor.13947.

## Data Availability

The data that support the findings of this study are available on request from the corresponding author. The data are not publicly available due to privacy or ethical restrictions.
